# Systematic review of the validity and reliability of consumer-wearable activity trackers

**DOI:** 10.1186/s12966-015-0314-1

**Published:** 2015-12-18

**Authors:** Kelly R. Evenson, Michelle M. Goto, Robert D. Furberg

**Affiliations:** Department of Epidemiology, Gillings School of Global Public Health, University of North Carolina—Chapel Hill, 137 East Franklin Street, Suite 306, Chapel Hill, 27514 NC USA; RTI International, Research Triangle Park, NC USA

**Keywords:** Distance, Energy expenditure, Fitbit, Intervention, Jawbone, Measurement, Physical activity, Sleep, Steps, Walking

## Abstract

**Background:**

Consumer-wearable activity trackers are electronic devices used for monitoring fitness- and other health-related metrics. The purpose of this systematic review was to summarize the evidence for validity and reliability of popular consumer-wearable activity trackers (Fitbit and Jawbone) and their ability to estimate steps, distance, physical activity, energy expenditure, and sleep.

**Methods:**

Searches included only full-length English language studies published in PubMed, Embase, SPORTDiscus, and Google Scholar through July 31, 2015. Two people reviewed and abstracted each included study.

**Results:**

In total, 22 studies were included in the review (20 on adults, 2 on youth). For laboratory-based studies using step counting or accelerometer steps, the correlation with tracker-assessed steps was high for both Fitbit and Jawbone (Pearson or intraclass correlation coefficients (CC) > =0.80). Only one study assessed distance for the Fitbit, finding an over-estimate at slower speeds and under-estimate at faster speeds. Two field-based studies compared accelerometry-assessed physical activity to the trackers, with one study finding higher correlation (Spearman CC 0.86, Fitbit) while another study found a wide range in correlation (intraclass CC 0.36–0.70, Fitbit and Jawbone). Using several different comparison measures (indirect and direct calorimetry, accelerometry, self-report), energy expenditure was more often under-estimated by either tracker. Total sleep time and sleep efficiency were over-estimated and wake after sleep onset was under-estimated comparing metrics from polysomnography to either tracker using a normal mode setting. No studies of intradevice reliability were found. Interdevice reliability was reported on seven studies using the Fitbit, but none for the Jawbone. Walking- and running-based Fitbit trials indicated consistently high interdevice reliability for steps (Pearson and intraclass CC 0.76–1.00), distance (intraclass CC 0.90–0.99), and energy expenditure (Pearson and intraclass CC 0.71–0.97). When wearing two Fitbits while sleeping, consistency between the devices was high.

**Conclusion:**

This systematic review indicated higher validity of steps, few studies on distance and physical activity, and lower validity for energy expenditure and sleep. The evidence reviewed indicated high interdevice reliability for steps, distance, energy expenditure, and sleep for certain Fitbit models. As new activity trackers and features are introduced to the market, documentation of the measurement properties can guide their use in research settings.

**Electronic supplementary material:**

The online version of this article (doi:10.1186/s12966-015-0314-1) contains supplementary material, which is available to authorized users.

## Background

Consumer wearable devices are a popular and growing market for monitoring physical activity, sleep, and other behaviors. The devices helped to grow what is known as the Quantified Self movement, engaging those who wish to track their own personal data to optimize health behaviors [1]. A subset of consumer wearable devices used for monitoring physical activity- and fitness-related metrics are referred to as “activity trackers” or “fitness trackers” [2]. Their popularity has risen as they have become more affordable, unobtrusive, and useful in their application. An activity tracker can provide feedback and offer interactive behavior change tools via a mobile device, base station, or computer for long-term tracking and data storage [3, 4]. The trackers enable self-monitoring towards daily or longer-term goals (such as a goal to walk a certain distance over time) and can be used to compare against one’s peers or a broader community of users, both of which are advantageous mediators to increasing walking and overall physical activity [3, 5].

A national United States (US) survey completed in 2012 indicated 69 % of adults tracked at least one health indicator for themselves, a family member, or friend using a tracking device (such as an activity tracker), paper tracking, or another method [6]. From this survey, 60 % of adults reported tracking weight, diet, or exercise. Those who tracked weight, diet, or exercise were similar by gender, but more likely to be non-Hispanic White or African American, older, and have at least a college degree compared to Hispanics, younger ages, and those with less than a college degree, respectively. Among those who tracked at least one health behavior or condition, 21 % used some form of technology to track the health data. Also among this group, 46 % indicated that tracking changed their overall approach to maintaining their health or the health of the person they cared for, 40 % indicated that it led them to ask a doctor new questions or obtain a second opinion, and 34 % indicated that it affected a decision about how to treat an illness or condition.

Activity trackers are being used not only in the consumer market but also in research studies. Physical activity-related interventions are using activity trackers for self-monitoring, reinforcement, goal-setting, and measurement (examples among adults [4, 7–11] and youth [12]). Before more widespread use of these trackers occurs in research studies, for either intervention or measurement purposes, it is important to establish their validity and reliability.

The purpose of this review was to summarize the evidence for validity and reliability of the most popular consumer-wearable activity trackers. Among a variety of trackers on the market, approximately 3.3 million sold between April 2013 to March 2014, with 96 % made by Fitbit (67 %), Jawbone (18 %), and Nike (11 %) [2]. Since Nike discontinued the sale of Fuelbands in 2014, our focus for this review was on activity trackers made by Fitbit and Jawbone. Before conducting the review, we searched company websites for documentation on the accuracy of measuring steps, distance, physical activity, energy expenditure, and sleep. The Fitbit company indicated that after multiple internal studies, they had “tuned the accuracy of the Fitbit tracker step counting functionality over hundreds of tests with multiple body types. All Fitbit trackers should be 95–97 % accurate for step counting when worn as recommended” [13]. However, no other information was provided to document the accuracy of steps, nor the other measures we reviewed. The Jawbone company indicated that “while variations in user, terrain, and activity conditions can influence specific calculations, testing has shown UP to provide industry-leading accuracy in tracking activity and sleep” [14]. Similarly, no other details were provided of how accuracy was determined. Therefore, we focused our search on the ability of these trackers to estimate steps, distance, physical activity, energy expenditure, and sleep. For each study included in the review, we also abstracted information on the tracker’s feasibility of use.

## Methods

### Literature search

Searches of PubMed, Embase, and SPORTDiscus were conducted to include only full-length studies published in English language journals through July 31, 2015. No start date was imposed in the search. If a publication was available online first before print, we attempted to obtain a copy; thus, some publications were officially published after July 31, 2015 but were available in the databases during our search period. Two separate searches were performed for the two activity trackers.

1. (Fitbit) AND (validity OR validation OR validate OR comparison OR comparisons OR comparative OR reliability OR accuracy)
2. (Jawbone) AND monitor AND (validity OR validation OR validate OR comparison OR comparisons OR comparative OR reliability OR accuracy)

The term “monitor” was added to the Jawbone search to reduce the number of dental-related articles retrieved. In addition, we reviewed Google Scholar similarly (same search terms, dates, only English language journals) and the reference lists of included studies for publications missed by the searches. We excluded abstracts (examples [15, 16]) and conference proceedings (example [17]). We also excluded studies focused on special populations, such as stroke and traumatic brain injury [18], chronic obstructive pulmonary disease [19], amputation [20], mental illness [21], or older adults in assisted living [22]. One study presented data on apparently healthy older adults without mobility impairments and those of similar ages with reduced mobility; therefore, we reported only on those without mobility impairments [23].

### Abstraction and analysis

First, we documented descriptive information on the activity trackers (models, release date, placement, size, weight, and cost) through internet searches conducted from May-July 2015. Second, an abstraction tool used for this review was expanded from a tool initially created by De Vries et al. [24] to document study characteristics and measurement properties of the activity trackers. Specifically, we extracted information on the study population, protocol, statistical analysis, and results related to validity, reliability, and feasibility. We also extracted any information provided by the studies on items entered into the activity tracker user account settings. A primary reviewer extracted details and a second reviewer checked each entry. Discrepancies in coding were resolved by consensus. For any abstracted information that was missing from the publication, we attempted to contact at least one author to obtain the information. Summary tables were created from the abstracted information.

Validity of the activity trackers included [25]:

- *Criterion validity*: comparing the trackers to a criterion measure of steps, distance traveled, physical activity, energy expenditure, and sleep.
- *Construct validity*: comparing the trackers to other constructs that should track or correlate positively (*convergent validity*) or negatively (*divergent validity*).

Reliability of the activity trackers included [25]:

- *Intradevice reliability*: test-retest results indicating consistency within the same tracker. This can be conducted in the lab (such as on a shaker table).
- *Interdevice reliability*: results indicating consistency across the same brand/type of tracker measured at the same time and worn in the same location. This can be assessed during activities performed in the laboratory or while free-living.

We interpreted the correlation coefficients (CC) using the following ratings: 0- < 0.2 poor, 0.2- < 0.4 fair, 0.4- < 0.6 moderate, 0.6- < 0.8 substantial, and 0.8- < 1.0 almost perfect [26]. Feasibility assessment included how much missing or lost data occurred and any feedback on wearing the trackers by participants.

## Results

>Through the systematic search, 67 records were identified, 39 were screened, and 22 were included in the review that reported on the validity or reliability of any Fitbit or Jawbone tracker. The Preferred Reporting Items for Systematic Reviews and Meta-Analyses (PRISMA) [27, 28] figure displays the detailed results from the search (Additional file 1). Twenty studies reported on at least one type of Fitbit tracker [15, 23, 29–46] and eight reported on at least one type of Jawbone tracker [30, 33, 35, 40, 42, 45, 47, 48].

### Fitbit tracker

The Fitbit company (San Francisco, CA; https://www.fitbit.com) has offered at least nine activity trackers since 2008 (Table 1). Depending on the type of activity tracker, the company recommends wearing them at the waist, wrist, pocket, or bra. The trackers contain a triaxial accelerometer and more recently an altimeter, heart rate, and global positioning system (GPS) monitor. Using proprietary algorithms, data from measures collected along with information input by the user can estimate steps, distance, physical activity, kilocalories, and sleep. Day-level data is summarized and available to the consumer. Minute-level data (called “intraday”) requires more effort to obtain, such as through the Fitbit API [32], and can be set at intervals of 1, 5, 10, 15, 20, or 60 min. Alternatively, data can be extracted using third-party service providers, such as Fitabase (Small Steps Labs LLC; https://www.fitabase.com), as was used in the study by Diaz et al. [15].

**Table 1:** Fitbit and Jawbone activity tracker characteristics (searched May-July 2015)

Tracker	Released date	Selected measures	Placement	Size (cm)	Weight (g)	Cost (US$)	Discontinuation
Fitbit							
Fitbit Classic (also referred to as the "original Fitbit" or "Fitbit Tracker")	September 2008	Steps, distance, calories, sleep	Waist, pocket, bra	5.5(h) × 1.9(w) × 1.4(d)	11	Not available	Winter 2012: discontinued
Fitbit Ultra	October 2011 (new hardware upgrade to the Classic)	Steps, distance, calories, sleep, altimeter	Waist, pocket, bra, wrist (requires Ultra sleep band)	5.5(h) × 1.9(w) × 1.4(d)	11	Not available	August 2012: discontinued
Fitbit One	September 2012 (update to the Ultra)	Steps, distance, calories, active minutes, sleep, altimeter	Waist, pocket, bra	4.8(h) × 1.9(w) × 1.0(d)	9	99.95	
Fitbit Zip	May 2013	Steps, distance, calories, active minutes	Waist, pocket, bra	3.6(h) × 2.9(w) × 1.0(d)	8	59.95	
Fitbit Flex	May 2013	Steps, distance, calories, active minutes, sleep	Wrist	Small: 14.0–17.6(c) × 1.4(w)	13	99.95	
				Large: 16.1–20.9(c) × 1.4(w)	15		
Fitbit Force	October 2013	Steps, distance, calories, active minutes, sleep, altimeter	Wrist	Small: 14.0–17.6(c) × 1.9(w)	31	Not available	February 2014: recalled by company because of skin reactions to the band
				Large: 16.1–20.9(c) × 1.9(w)			
Fitbit Charge	November 2014	Steps, distance, calories, active minutes, altimeter, sleep	Wrist	Small: 14.0–17.0(c) × 2.1(w)	23	129.95	
				Large: 16.1–20.0(c) × 2.1(w)			
				Extra Large: 19.8–23.0(c) × 2.1(w)			
Fitbit Surge	January 2015	Steps, distance, calories, active minutes, altimeter, sleep, heart rate, GPS	Wrist	Small: 14.0–16.0(c) × 3.4(w)	77	249.95	
				Large: 16.0–19.8(c) × 3.4(w)			
				Extra Large: 19.8–22.6(c) × 3.4(w)			
				Small: 14.0–17.0(c) × 2.1(w)			
Fitbit Charge HR	January 2015	Steps, distance, calories, active minutes, altimeter, sleep, heart rate	Wrist	Large: 16.1–19.4(c) × 2.1(w)	23	149.95	
				Extra Large: 19.4–23.0(c) × 2.1(w)			
Jawbone							
Jawbone UP	November 2011	Steps, calories, distance (app), sleep	Wrist	Small: 14.0–15.5	19	99.99	December 2011: company provided refunds because the band had trouble holding a charge and synching to the band hardware
				Medium: 15.5–18.0	21		
				Large: 18.0–20.0	23		
Jawbone UP24	November 2013	Steps, calories, distance (app), sleep	Wrist	Small: 5.2(w) × 3.5(h) (inner); 6.6(w) × 5.0(h) (outer)	19	129.99	July 2015: no longer for sale on the company's website
				Medium: 6.3(w) × 4.0(h) (inner); 7.6(w) × 5.4(h) (outer)	22		
				Large: 6.9(w) × 4.3(h) (inner); 8.1(w) × 5.6(h) (outer)	23		
Jawbone UP MOVE	November 2014	Steps, calories, distance (app), sleep	Waist, pocket, bra, wrist (requires separate wrist strap)	2.8(diameter) × 1.0(d)	7	49.99	
Jawbone UP2	April 2015	Steps, calories, distance (app), sleep	Wrist	14.0–19.0(c) × 1.2(w)	25	99.99	
Jawbone UP3	November 2014	Steps, calories, distance (app), sleep, bioimpedance (heart rate, respiration, galvanic skin response), skin and ambient temperature	Wrist	14.0–19.0(c) × 1.2(w)	29	179.99	
Jawbone UP4	July 2015	Steps, calories, distance (app), sleep, bioimpedance (heart rate, respiration, galvanic skin response), skin and ambient temperature	Wrist	14.0–19.0(c) × 1.2(w)	29	199.99	

Abbreviations: *c* circumference, *d* depth, *GPS* global positioning system, *h* height, *w* width

The Fitbit One updated the Fitbit Ultra in 2012, which in turn updated the Fitbit Classic in 2011, and all three are shaped similarly as a clip. The Fitbit Zip is teardrop-shaped and the Fitbit Flex is designed for the wrist. The following Fitbit trackers were explored for validity (Table 2):

1. Classic worn at the waist [29, 31, 39, 41] and non-dominant wrist [38];
2. Ultra worn at the waist/hip [23, 29, 34, 36, 40], pants pocket [32, 36], dominant-handed wrist [23], non-dominant wrist [37], shirt collar [36], and bra [36];
3. One worn at the waist [15, 30, 32, 33, 35, 42, 43, 46], pants pocket [43], and ankle [46];
4. Zip worn at the waist [30, 33, 35, 44]; and
5. Flex worn on the wrist [15, 30, 45].

**Table 2:** Fitbit and Jawbone studies of interdevice reliability and validity (listed by author's last name and publication year)

	Interdevice reliability					Validity				
Motion sensor	Steps	Distance	Physical activity	Energy expenditure	Sleep	Steps	Distance	Physical activity	Energy expenditure	Sleep
Fitbit										
Fitbit Classic (also referred to as the "original Fitbit" or "Fitbit Tracker")	Adam Noah 2013 [29]			Adam Noah 2013 [29]	Montgomery- Downs 2012 [38]	Adam Noah 2013 [29]			Adam Noah 2013 [29]; Dannecker 2013 [31]: Sasaki 2015 [39]; Stahl 2014 [41]	Montgomery- Downs 2012 [38]
Fitbit Ultra	Adam Noah 2013 [29]; Dontje 2015 [32]; Mammen 2012 [36]			Adam Noah 2013 [29]	Meltzer 2015 [37]	Adam Noah 2013 [29]; Gusmer 2014 [34]; Lauritzen 2013 [23]; Mammen 2012 [36]; Stackpool 2014 [40]			Adam Noah 2013 [29]; Gusmer 2014 [34]; Stackpool 2014 [40]	Meltzer 2015 [37]
Fitbit One	Diaz 2015 [15]; Takacs 2014 [43]	Takacs 2014		Diaz 2015 [15]		Case 2015 [30]; Diaz 2015 [15]; Ferguson 2015 [33]; Simpson 2015 [46]; Storm 2015 [42]; Takacs 2014 [43]	Takacs 2014 [43]	Ferguson 2015 [33]	Diaz 2015 [15]; Ferguson 2015 [33]; Lee 2014 [35]	Ferguson 2015 [33]
Fitbit Zip						Case 2015 [30]; Ferguson 2015 [33]; Tully 2014 [44]		Ferguson 2015 [33]; Tully 2014 [44]	Ferguson 2015 [33]; Lee 2014 [35]	
Fitbit Flex	Diaz 2015 [15]			Diaz 2015 [15]		Case 2015 [30]; Diaz 2015 [15]			Bai 2015 [45]; Diaz 2015 [15]	
Jawbone										
Jawbone UP						Ferguson 2015 [33]; Stackpool 2014 [40]; Storm 2014 [42]		Ferguson 2015 [33]	Ferguson 2015 [33]; Lee 2014 [35]; Stackpool 2014 [40]	de Zambotti 2015a [47]; de Zambotti 2015b; Ferguson 2015
Jawbone UP24						Case 2015 [30]				Bai 2015 [45]

We found no studies for the Fitbit Force, Surge, Charge, or Charge HR, or the Jawbone UP MOVE, UP2, UP3, or UP4

Reliability studies included the Classic worn at the waist [29] and non-dominant wrist [38]; the Ultra worn at the waist/hip [29, 36], pants pocket [32], and non-dominant wrist [37]; the One worn at the waist [15, 43] and pants pocket [43]; and the Flex worn on the wrist [15].

### Jawbone tracker

The Jawbone company (San Francisco, CA; https://jawbone.com) has offered at least six activity trackers since 2011 (Table 1). Their trackers are worn at the wrist, with the exception of the UP MOVE tracker to be worn at the waist, pocket, or bra. The trackers contain a triaxial accelerometer, collecting data at 30 Hertz, and more recently bioelectrical impedance (for heart rate, respiration, and skin response), as well as both skin and ambient temperatures. Using proprietary algorithms, data from measures collected along with information input by the user can estimate steps, distance, physical activity, kilocalories, and sleep. Currently, only day-level data is available to the consumer.

The following two Jawbone trackers, both designed for the wrist, were explored for validity (Table 2):

1. UP worn on the wrist [33, 35, 40, 42, 47, 48] and
2. UP24 worn on the wrist [30, 45].

No Jawbone trackers were explored for reliability.

About half of the studies reported the data entered into the tracker user account [29, 33–35, 39, 41, 43], which was usually age, gender, height, and weight. One study also reported entering stride length [34], another study input handedness and smoking status [35], and another study used event markers to denote when an activity started and ended [39]. A sleep study indicated that they manually switched the band from active to sleep mode in conjunction with lights on/off [48]. Other studies did not report what data were input into the user account [15, 23, 30–32, 36–38, 40, 42, 44–47].

#### Description of studies

Data collection was primarily conducted in the US, with one or two studies conducted in Australia [33], Canada [36, 43, 46], the Netherlands [32], Northern Ireland [44], Spain [23], and the United Kingdom [42] (Table 3). Studies usually included an apparently healthy sample and, where reported, almost all participants had a normal body mass index (BMI). Additionally, participants were > =18 years and mostly younger to middle age, except for one study focusing exclusively on adults > =60 years [41] and two studies on youth [37, 48]. Data were collected between 2010 [38] to 2015 [47].

**Table 3:** Characteristics of studies included in the systematic review (listed by author's last name and publication year)

Author (year)	Location of lab or recruitment area	Sample size (for validity and reliability studies)	Mean age (SD), range	Mean body mass index (SD), range in kilograms/ meters squared	Data collection year(s)	Inclusion criteria
Adam Noah (2013) [29]	Northeastern university, US	16 and 23 (V and R)	26.7 (7.6)	Not reported	2011-2012	Apparently healthy participants, had to participate in moderate to vigorous physical activity based on the International Physical Activity Questionnaire (> = 150 minutes/week of moderate intensity or > =75 minutes/week of vigorous intensity)
Bai (2015) [45]	Ames, Iowa, US	52 (V)	18–65	24.0, 17.6–39.9	2014	Apparently healthy adults with no major surgeries in the past year
Case (2015) [30]	Philadelphia, Pennsylvania, US	14 (V)	28.1 (6.2)	22.7 (1.5)	2014	Apparently healthy adults
Dannecker (2013) [31]	Fort Collins and Denver, Colorado, US	19 (V)	26.9 (6.6)	25.1 (4.6)	2010	Apparently healthy adults, inactive to moderately active (<6 hours/week of exercise)
de Zambotti (2015a) [47]	San Francisco, California, US	28 (V)	50.1 (3.9)	24.6 (3.6)	2014–2015	Perimenopausal women
de Zambotti (2015b) [48]	San Francisco, California, US	65 (V)	15.8 (2.5)	21.2 (3.5)	2014	Apparently healthy without sleep disorders
Diaz (2015) [15]	New York City, New York, US	23 (V and R)	20–54	19.6–29.9	2013–2014	Apparently healthy
Dontje (2015)[32]	Groningen, The Netherlands	1 (R)	46	Not reported	2012	Not reported
Ferguson (2015) [33]	Adelaide, South Australia	21 (V)	32.8 (10.2), 20–59	27.3 (3.2) male; 25.5 (5.2) female	2013	Apparently healthy
Gusmer (2014) [34]	Minneapolis, Minnesota, US	32 (V)	21.1 (1.7), 18–29	Not reported	2012	Apparently healthy
Lauritzen (2013) [23]	Seville, Spain	6 (V)	35.3 (6.5), 24–45	Not reported	not reported	Not reporting on sample with reduced mobility and no results on older sample with normal mobility
Lee (2014) [35]	Ames, Iowa, US	60 (V)	24.2 (4.7) female; 28.6 (6.4) male	24.3 (2.6), 19.528.0 male; 21.8 (2.7), 18.1–31.2 female	2013	No major disease and nonsmokers
Mammen (2012) [36]	Toronto, Canada	10 (V)and 1 (R)	23.0 (1.2), 20–25	21.4 (1.9)	2011–2012	Healthy young adults
Meltzer (2015) [37]	Birmingham, Alabama, US	63 (V) and 9 (R)	9.7 (4.6), 3–17	Not reported	2012–2013	Sample referred to clinic for sleep disordered breathing; results of polysomnography indicated: 61 % none, 23 % mild, 16 % moderate to severe
Montgomery-Downs (2012) [38]	Morgantown, West Virginia, US	24 (V) and 3 (R)	26.1, 19–41	Not reported	2010	Healthy adults, no sleep disorders
Sasaki (2015) [39]	Amherst, Massachusetts, US	20 (V)	24.1 (4.5)	23.9 (2.9)	2011–2012	Apparently healthy
Simpson (2015) [46]	Vancouver, Canada	42 (V)	73 (6.9)	26.1 (4.6)	2014	> = 65 years, able to walk unassisted
Stackpool (2014) [40]	LaCrosse, Wisconsin, US	20 (V)	18–44	Not reported	2013	Healthy volunteers; all were recreationally active (2–5 hours/week)
Stahl (2014) [41]	Morgantown, West Virginia, US	10 (V)	63.8 (3.2), 60–68	24.5 (4.2)	2011	None noted; on average participants reported 3 chronic health conditions, no functional limitations, and rated their health as "good"
Storm (2015) [42]	Sheffield, United Kingdom	16 (V)	28.9 (2.7)	23.5 (2.3)	2013	No reported impairment or morbidity that could interfere with physical activity assessment
Takacs (2014) [43]	Vancouver, Canada	30 (V and R)	29.6 (5.7)	22.7 (3.0)	2013	Able to walk on a treadmill for 30 min; no neurological, cognitive or musculoskeletal disorders
Tully (2014) [44]	Belfast, Northern Ireland	42 (V)	43	Not reported	2013	Apparently healthy staff of Queen's University Belfast

Abbreviations: *R* reliability sample size, *SD* standard deviation, *US* United States, *V* validity sample size

#### Validity

All but one study (21/22) explored the validity of at least one type of activity tracker (Table 4). Sample sizes of the studies ranged from six [23] to 65 [48]. For any Fitbit tracker, validity was reported from 12 studies on steps [15, 23, 29, 30, 33, 34, 36, 40, 42–44, 46], one study on distance [43], two studies on physical activity [33, 44], ten studies on energy expenditure [15, 29, 31, 33–35, 39–41, 45], and three studies on sleep [33, 37, 38] (Table 2). For any Jawbone tracker, validity was reported from four studies on steps [30, 33, 40, 42], zero studies on distance, one study on physical activity [33], three studies on energy expenditure [33, 35, 40], and three studies on sleep [33, 47, 48]. The following sections detail the validity results for each of the five measures.

**Table 4:** Fitbit and Jawbone validity studies (listed by author's last name and publication year)

	Sample characteristics		Tracker wearing protocol			Measurements			Validity results
Author (year)	*n*	% female	Activity	Lab/field	Validity criterion (measure assessed)	Type	Placement	Measures	
Adam Noah et. al (2013) [29]	16	38	6 min each of treadmill walking (3.5 mph), walking with incline (3.5 mph at 5 %), jogging (5.5 mph), and stair stepping (30.5 centimeter step at 96 beats/min)	Lab	Two Actical accelerometers (steps), indirect calorimetry using K4b2 Cosmed (EE)	Ultra (Fitbit)	Waist (one on each side)	Steps/min, kilocalories/min	Fitbit Ultra vs. Actical ICC: average 0.94, range 0.80–0.99 (steps); Fitbit Ultra vs. Cosmed ICC: average 0.77, range 0.58-0.87 (kilocalories)
	23	43				Classic (Fitbit)	Waist (one on each side)	Steps/min, kilocalories/min	Fitbit vs. Actical ICC: average 0.93, range 0.82–0.98 (steps); Fitbit vs. Cosmed ICC: average 0.74, range 0.18-0.72 (kilocalories)
Bai et. al (2015) [45]	52	46	20 min sedentary, 25 min treadmill at self-selected speed, 25 min resistance exercise	Lab	Indirect calorimetry using Oxycon Mobile (EE)	Flex (Fitbit)	Left wrist	Kilocalories/80- min trial	Overestimated overall EE by 20.4 kilocalories; Pearson CC 0.78; overall mean absolute error 16.8 %
						UP24 (Jawbone)	Right wrist		Underestimated overall EE by 23.1 kilocalories; Pearson CC 0.77; overall mean absolute error 18.2 %
Case et. al (2015) [30]	14	71	Treadmill walking at 3.0 mph for 500 and 1500 steps, each done twice	Lab	Tally counter (steps)	One (Fitbit)	Waist	Steps/trial	500 step trial (*n* = 27 observations) mean 498.6 (SD 3.7); 1500 step trial (*n* = 26 observations) mean 1497.0 (SD 10.7)
						Zip (Fitbit)	Waist	Steps/trial	500 step trial (*n* = 27 observations) mean 498.6 (SD 10.8); 1500 step trial (*n* = 27 observations) mean 1498.4 (SD 10.4)
						Flex (Fitbit)	Wrist	Steps/trial	500 step trial (*n* = 28 observations) mean 465.4 (SD 92.1); 1500 step trial (*n* = 28 observations) mean 1378.0 (SD 142.7)
						UP24 (Jawbone)	Wrist	Steps/trial	500 step trial (*n* = 28 observations) mean 477.5 (SD 102.1); 1500 step trial (*n* = 28 observations) mean 1477.0 (SD 174.4)
Dannecker et. al (2013) [31]	19 (16 with Fitbit data)	47 (from *n* = 19)	Resting, supine, sitting, standing, free living activity, and 6 random activities out of 8 (walking (2.5 mph, 3.5 mph, or 2.5 mph with 2.5 % grade), stepping, sweeping, cycling (75 watts), standing, sitting	Lab	4 h stay in whole room calorimeter (EE)	Classic (Fitbit)	Belt at anterior superior iliac spine	Total EE during the 3.5-h period while in the room calorimeter (omitted first 30 minutes)	Root-mean-square error of tracker 28.7 % or 143 kilocalories; root-mean-square error of tracker after labeling activities 12.9 % or 64 kilocalories
de Zambotti et. al (2015a) [47]	28	100	One nights sleep (*n* = 10), 2 nights sleep (*n* = 18)	Lab	Polysomnography (sleep)	UP (Jawbone)	Non dominant wrist	TST, sleep onset latency, WASO	Overestimated TST by 26.6 ± 35.3 min (*p* < 0.001) and sleep onset latency by 5.2 ± 9.6 min (*p* = 0.005); underestimated WASO by 31.2 ± 32.3 min (*p* < 0.001)
de Zambotti et. al (2015b) [48]	65	43	One nights sleep	Lab	Polysomnography (sleep)	UP (Jawbone)	Non dominant wrist	TST, sleep efficiency, sleep onset latency, WASO	Overestimated TST by 10.0 min (*p* < 0.001), sleep efficiency by 1.9 % (*p* < 0.001), and sleep onset latency by 1.3 min (*p* = 0.33); underestimated WASO by 10.6 min (*p* < .001)
Diaz et. al (2015) [15]	23	57	6 min each of treadmill walking (1.9 mph, 3.0 mph, 4.0 mph) and jogging (5.2 mph)	Lab	Counting from a video recording (steps), indirect calorimetry using Ultima CPX (EE)	One (Fitbit)	2 on right hip, 1 on left hip	Steps/min, kilocalories/min	Pearson CC 0.97–0.99 and mean difference −3.1 to −0.3 (steps); Pearson CC 0.86-0.87 (kilocalories) and mean difference −0.8 to 0.4 kilocalories
						Flex (Fitbit)	1 on each wrist	Steps/min, kilocalories/min	Pearson CC 0.77-0.85 and mean difference −26.3 to −2.9 (steps); Pearson CC 0.88 and mean difference −0.2 to 2.6 (kilocalories)
						One (Fitbit)	Right hip	Steps/day, MVPA min/day, kilocalories/day, sleep min/day	Pearson CC 0.99 (steps), 0.91 (MVPA), 0.76 (kilocalories), 0.92 (sleep); ICC 0.95 (steps), 0.46 (MVPA), 0.55 (kilocalories), 0.90 (sleep); mean absolute difference 779 (steps), 58.6 (MVPA), 349 (kilocalories), 23.0 (sleep); range of differences = −890 to 1849 (steps), 1.0 to 137.2 (MVPA), −1724 to −83 (kilocalories), 45 to 76 (sleep)
						Zip (Fitbit)	Right hip	Steps/day, MVPA min/day, kilocalories/day	Pearson CC 0.99 (steps), 0.88 (MVPA), 0.81 (kilocalories); ICC 0.98 (steps), 0.36 (MVPA), 0.57 (kilocalories); mean absolute difference 447 (steps), 89.8 (MVPA), 484 (kilocalories); range of differences −970 to 1596 (steps), 10.0 to 157.2 (MVPA), −1145 to 218 (kilocalories)
Ferguson et. al (2015) [33]	21	52	48 h (including sleep, excluding showering) of free-living conditions, no activity restrictions/guidelines	Field	BodyMedia SenseWear model MF (steps, physical activity, EE, sleep); ActiGraph GT3X+ (steps, physical activity)	UP (Jawbone)	Left wrist	Steps/day, MVPA min/day, kilocalories/day, sleep min/day	Pearson CC 0.97 (steps), 0.81 (MVPA), 0.74 (kilocalories), 0.89 (sleep); ICC 0.97 (steps), 0.70 (MVPA), 0.27 (kilocalories), 0.85 (sleep); mean absolute difference 806 (steps), 18.0 (MVPA), 866 (kilocalories), 22.0 (sleep); range of differences −1978 to 2252 (steps), −4.7 to 96.5 (MVPA), −1937 to −94 (kilocalories), − 31 to 132 (sleep)
Gusmer et. al (2014) [34]	32	78	30-min phases of treadmill walking at slow and brisk speeds (±10 % of selfselected comfortable walking speed)	Lab	ActiGraph G1TM (steps), CPX Ultima metabolic cart (EE)	Ultra (Fitbit)	Right hip	Steps/min, kilocalories/trial	Pearson CC: slow walk: 0.97 (steps: mean 105.3 ActiGraph vs. 105.9 Ultra), 0.69 (kilocalories: mean 100.9 cart vs. 88.0 Ultra); brisk walking: 0.996 (steps: mean 114.2 ActiGraph vs. 113.9 Ultra), 0.94 (kilocalories: mean 121.9 cart vs. 100.9 Ultra)
Lauritzen et. al (2013) [23]	6	0	20-meter walk at participant's normal pace	Lab	Counting from a video recording (steps)	Ultra (Fitbit)	1 on belt/pants pocket on dominant leg, 1 on wrist of dominant hand	Steps/20-min trial	Hip error 2.9 % (SD 2.3 %); wrist error 31.3 % (SD 30.7 %)
						One (Fitbit)	Waist	Kilocalories/trial	Mean absolute error 10.4 %; Pearson CC 0.81; root-mean-square error 40.1; did not fall in 90 % equivalence interval; systematic bias with slope −0.22 comparing One (x) to Oxycon (y); Pearson CC to ActiGraph 0.80
Lee et. al (2014) [35]	60	50	13 activities that were all 5 min in length except for treadmill (3 min each) totalling 69 minutes	Lab	Oxycon Mobile (EE); ActiGraph GT3X+ worn on hip, applied Sasaki et al. 2011 [39] algorithm (EE)	Zip (Fitbit)	Waist	Kilocalories/trial	Mean absolute error 10.1 %; Pearson CC 0.81; root-mean-square error 40.8; fell within 90 % equivalence interval from measured EE; systematic bias with slope - 0.29 comparing Zip (x) to Oxycon (y); Pearson CC to ActiGraph 0.77
						UP (Jawbone)	Left wrist	Kilocalories/trial	Mean absolute error 12.2 %; Pearson CC 0.74; root-mean-square error 45.8; did not fall in 90 % equivalence interval; no systematic direction of bias with slope - 0.03 comparing UP (x) to Oxycon (y); Pearson CC to ActiGraph 0.65
Mammen et. al (2012) [36]	10	50	One min on the treadmill at each of 8 speeds (4 walking and 4 running)	Lab	Manually count (steps)	Ultra (Fitbit)	Waist, inside the pants pocket, shirt collar (men) or bra (women)	Steps/trial	Waist-worn Ultra under counted at 2 km/hour (31 steps/min; *p* < 0.05) but had similar counts at > =3 km/hour. Pocket- worn Ultra under counted during running (10, 19, 34, 38 steps/min at 8, 9, 10, and 11 km/hour, respectively; *p* < 0.05), but recorded similar counts when walking (2, 3, 4.5, and 6 km/hour). Similar counts across walk/run trials for collar-(males) or bra-(females) worn Ultras.
Meltzer et. al (2015) [37]	63	51	One night's sleep	Lab	Polysomnography (sleep)	Ultra (Fitbit)	Non dominant wrist	TST, sleep efficiency, WASO	Normal mode overestimated TST by 41 minutes and sleep efficiency by 8 %, underestimated WASO by 32 minutes; 87 % sensitivity, 52 % specificity, 84 % accuracy. Sensitive mode underestimated TST by 105 minutes and sleep efficiency by 21 % and overestimated WASO by 106 minutes; 70 % sensitivity, 79 % specificity, 71 % accuracy.
Montgomery- Downs et. al (2012) [38]	24	40	One night's sleep	Lab	Polysomnography (sleep)	Classic (Fitbit)	Non dominant wrist	TST, sleep efficiency	Polysomnography recorded 465.0 min (SD 48.4) with 79.5 % sleep efficiency and 370.9 min (SD 70.3) TST; Fitbit measured 94.0 % sleep efficiency and 438.0 min TST; Fitbit overestimated sleep efficiency compared to polysomnography by 14.5 % (SD 10.7 %) and overestimated TST by mean 67.1 min (SD 51.3).
Sasaki et. al (2015) [39]	20	50	Visit 1: 6 min each of treadmill walking (3.0 at 5 % and 4.0 at 5 %) and jogging (5.5 mph), three trials; visit 2: 6 min each of household activities (choice from 3 activity routines)	Lab	Oxycon Mobile (EE)	Classic (Fitbit)	Belt around waist in line with the anterior axillary line	Total EE (rest plus activity)	Pearson CC 0.86; systematic underestimation of EE by the Fitbit with a mean bias of −4.5 ± 1.0 kcals/6 min; for 6 of 15 activities the Fitbit significantly underestimated EE (stairs, cycling, laundry, raking, treadmill 3.0 mph with 5 % grade, treadmill 4.0 mph with 5 % grade) and 1 of 15 activities the Fitbit significantly overestimated EE (carrying groceries)
Simpson et. al (2015) [46]	42	74	8 trials of walking 15 meters (self selected speed and 0.3-0.9 m/s at 0.1 increments)	Lab	Counting from a video recording (steps)	One (Fitbit)	Right waist, right ankle	Steps/trial	% error: 0.3 m/s: ankle 14.5, waist 98.4; 0.4 m/s: ankle 5.9, waist 82.0; 0.5 m/s: ankle 4.1, waist 40.4; 0.6 m/s: ankle 3.2, waist 21.6; 0.7 m/s: ankle 2.5, waist 10.5; 0.8 m/s: ankle 2.8, waist 7.0; 0.9 m/s: ankle 2.8, waist 5.6; Bland Altman mean difference −0.4 to 5.7 steps for ankle and 1.4 to 48.0 for waist
Stackpool et. al (2014) [40]	20	50	20 min each of: treadmill walking, treadmill running, elliptical cross-training, agility-related exercises	Lab	Manually counting (steps); Oxycon Mobile (EE)	Ultra (Fitbit)	Hip	Steps and kilocalories for each 20-min bout	Pearson CC: treadmill walking (0.99 steps, 0.24 kilocalories), treadmill running (0.44 steps, 0.63 kilocalories), elliptical (0.99 steps, 0.47 kilocalories), agility (0.47 steps, 0.67 kilocalories)
						UP (Jawbone)	Wrist	Steps and kilocalories for each 20-min bout	Pearson CC: treadmill walking (0.98 steps, 0.87 kilocalories), treadmill running (0.98 steps, 0.69 kilocalories), elliptical (0.99 steps, 0.40 kilocalories), agility (0.34 steps, 0.65 kilocalories)
Stahl and Insana (2014) [41]	10	30	During waking hours for 10 consecutive days	Field	Self-reported estimation of expended kilocalories/week from CHAMPS questionnaire (EE). Note: kilocalories/week divided by 7 to obtain kilocalories/day; then basal metabolic rate was added to the kilocalories/day.	Classic (Fitbit)	Waist	Kilocalories/day	Pearson CC 0.61; Fitbit underestimated by a mean of 195.0 kilocalories/day; 70 % of participant's data were within 1 SD and 100 % were within 2 SD
Storm et. al (2015) [42]	16	38	11-min walking protocol (included indoor and outdoor walking and steps) repeated at self-selected natural, slow, and fast speeds	Lab	OPAL sensors placed on each ankle (steps)	One (Fitbit)	Left waist	Steps/11-min trial	1.1 % self-selected walk, 1.0 % fast walk; limits of agreement 15 ± 35 steps; under estimated for slow walk (−25 mean steps), self-selected walk (−12 mean steps), fast walk (−9 mean steps)
						UP (Jawbone)	Right wrist	Steps/11-min trial	Mean absolute error 10.1 % slow walk, 2.5 % self-selected walk, 2.1 % fast walk; limits of agreement 16 ± 135; under estimated for slow walk (−35 mean steps), self-selected walk (−4 mean steps), fast walk (−9 mean steps)
Takacs et. al (2014) [43]	30	50	5 min each of treadmill walking (0.90, 1.12, 1.33, 1.54, 1.78 meters/second)	Lab	Motion capture system and manually counting (steps); treadmill output (distance)	One (Fitbit)	1 right hip, 1 left hip, 1 in front pocket of the dominant leg	Steps/trial, distance/trial	Steps: no significant difference (*p* > 0.05) between observed and One step counts at any of the 3 locations, ICC 0.97-1.00, relative error <1.3 %. Distance: significant differences between observed and One distance, ICC 0.0-0.05, relative error 5.0-39.6 %.
Tully et. al (2014) [44]	42	60	7 days of free-living wear excluding water activities and sleep	Field	ActiGraph GT3X and Yamax CW700 pedometer (steps, physical activity)	Zip (Fitbit)	Right waist	Steps/day, MVPA min/day	Spearman CC: 0.91 (ActiGraph steps), 0.86 (ActiGraph MVPA), 0.91 (Yamax steps)

Abbreviations: *CC* correlation coefficient, *CHAMPS* Community Healthy Activities Model Program for Seniors, *EE* energy expenditure, *ICC* intraclass correlation coefficient, *km* kilometers, *m* meters, *m/s* meters/second, *min* minute, *mph* miles per hour, *MVPA* moderate to vigorous physical activity, *SD* standard deviation, *TST* total sleep time, *WASO* wake after sleep onset

### Validity for steps

The criterion measures for counting steps included comparisons against manual step counting, either in-person [30, 36, 40] or with video recording [15, 23, 43, 46], or steps recorded by pedometers (Yamax CW-700 [44]) or accelerometers (Actical [29], ActiGraph GT1M [34], ActiGraph GT3X [44], ActiGraph GT3X+ [33], Body Media SenseWear [33], and Opal sensors [42]). Hip-worn trackers generally outperformed wrist-worn trackers for step accuracy [15, 23, 30, 40]. One study found less error for the ankle-worn One compared to the waist-worn One [46].

For laboratory-based studies using step counting as the criterion [15, 23, 43], correlation with steps from the tracker was generally high (if reported, the mean correlations were > =0.80) for the Ultra (for most treadmill speeds [36]; for treadmill walking and elliptical but not for running or agility drills [40]), One [30, 43], Zip [30], and UP (for treadmill walking, running, and elliptical [40]) trackers. However, several studies indicated that the One [15], Flex [15, 30], Ultra (waist worn at slower walking speed (2 km/h) and the pocket worn at faster speeds (> = 8 km/h)) [36]), and UP24 [30] under-estimated steps during treadmill walking and running.

For studies using accelerometry as the criterion, correlation with tracker steps was also generally high (if reported, the mean correlations were > =0.80) for the Classic [29], Ultra [29, 34], Zip [44], One [33], and UP [33] trackers. However, several studies indicated that the One [42], Flex [15, 30], UP [33](at slow walking speeds [42]), and UP24 [30] under-estimated steps during treadmill walking and running. In contrast, in a study of 21 participants wearing the One for 2 days without restrictions, compared to an accelerometer the tracker generally over-counted steps for the One (mean absolute difference 779 steps/day) [33]. In one free-living study, the researcher wore both the Ultra and a Yamax pedometer while seated in a car driving on paved roads for about 20 min [36]. During this time no steps were recorded for the Ultra, while the pedometer recorded three steps.

### Validity for distance

Only one study explored the validity of distance walked using the treadmill distance as the criterion. Among 30 participants, they found that the hip- and pocket-worn One generally over-estimated distance at the slower speeds (0.90–1.33 m/s), but under-estimated at faster speeds (1.78 m/s) [43].

### Validity for physical activity

The criterion measures for two studies exploring physical activity relied on other accelerometers (ActiGraph GT3X [44] and ActiGraph GT3X+ [33], both using Freedson et al. cutpoints [49], and Body Media SenseWear [33]). Based on 42 participants wearing the Zip for 1 week during waking hours, moderate-to-vigorous physical activity showed almost perfect correlation with an accelerometer (Spearman CC 0.86) [44]. However, in another study of 21 participants wearing the Zip, One, and UP for 2 days without restrictions, compared to an accelerometer the trackers generally over-counted minutes of moderate-to-vigorous physical activity (mean absolute difference 89.8, 58.6, 18.0 min/day, respectively and intraclass CC 0.36, 0.46, 0.70, respectively) [33].

### Validity for energy expenditure

The criterion measures for energy expenditure assessed in kilocalories was indirect calorimetry [15, 29, 34, 35, 39, 40, 45], direct calorimetry [31], accelerometry (ActiGraph GT3X+ with a conversion equation [50] to estimate kilocalories [35] and BodyMedia SenseWear [33]), and self-reported data using a questionnaire [41]. Generally, regardless of the criterion used, energy expenditure was under-estimated for the Classic [29, 31, 39, 41], One [33, 35], Flex, Ultra [29, 34] (for running, elliptical, and agility drills [40]), Zip [33, 35], UP [33, 35](for agility drills [40]), and UP24 [45]. When correlations were reported, they ranged widely [15, 29, 34, 35, 45]. A few studies indicated energy expenditure was over-estimated compared to indirect calorimetry: the Ultra during walking [40], the Zip across a variety of laboratory-based activities [35], the Flex during several combined activities (sedentary, aerobic, and resistance exercises) [45], and the UP during running [40].

### Validity for sleep

Five studies explored the validity of sleep measures, four using polysomnography (PSG) [37, 38, 47, 48] and the other using the BodyMedia SenseWear device [33] as the criterion. Compared to PSG, the Classic [38], Ultra [37], and UP [47, 48] over-estimated total sleep time and sleep efficiency and under-estimated wake after sleep onset, resulting in high sensitivity and poor specificity. However, for the Ultra when using the sensitive mode setting, total sleep time and sleep efficiency were under-estimated and wake after sleep onset was over-estimated. In a study of 21 adults wearing the One and UP for 2 days without restrictions, compared to an accelerometer the trackers generally over-estimated time in sleep (mean absolute difference 23.0, 22.0 min/day, respectively and intraclass CC 0.90, 0.85, respectively) [33].

#### Reliability

No study reported on the intradevice or interdevice reliability of the Jawbone, or the intradevice reliability of the Fitbit. Seven studies reported on the interdevice reliability of several Fitbit trackers (Table 5), with sample sizes ranging from one [32, 36] to 30 [43]. Four studies were laboratory-based focusing solely on locomotion on the treadmill [15, 29, 36, 43], two studies were laboratory-based requiring monitoring with a PSG [37, 38], and one study was field-based [32]. For any Fitbit tracker, interdevice reliability was reported from five studies on steps [15, 29, 32, 36, 43], one study on distance [43], no studies on physical activity, two studies on energy expenditure [15, 29], and two studies on sleep [37, 38]. The following sections detail the reliability results for each of the five measures.

**Table 5:** Fitbit and Jawbone reliability studies (listed by author's last name and publication year)

	Sample characteristics		Tracker wearing protocol		Measurements			Interdevice reliability results
Author (year)	*n*	% female	Activity	Lab/field	Type	Placement	Measures	
Adam Noah et. al (2013) [29]	16	38	Treadmill walking (3.5 mph), walking with incline (3.5 mph at 5 %), jogging (5.5 mph), and stair stepping (30.5 centimeter step at 96 beats/min)	Lab	Ultra (Fitbit)	Waist (1 on each side)	Steps/min, kilocalories/min	ICC comparing 2 different devices worn at once: range 0.76-0.99 (steps), range 0.91-0.97 (kilocalories)
	23	43			Classic (Fitbit)	Waist (1 on each side)	Steps/min, kilocalories/min	Comparing 2 different devices worn at once: ICC = average 0.88, range 0.86-0.91 (steps); average 0.87, range 0.74-0.92 (kilocalories)
Diaz (2015) [15]	23	57	6 min each of treadmill walking (1.9 mph, 3.0 mph, 4.0 mph) and jogging (5.2 mph)	Lab	One (Fitbit)	2 on right hip, 1 on left hip	Steps/min, kilocalories/min	Pearson CC left and right hips: 0.99 (steps), 0.97 (kilocalories); Pearson CC two right hip devices: 0.99 (steps), 0.96 (kilocalories)
					Flex (Fitbit)	1 on each wrist	Steps/min, kilocalories/min	Pearson CC left and right wrists: 0.90 (steps), 0.95 (kilocalories)
Dontje (2015) [32]	1	0	8 consecutive days excluding sleep and water-based activities	Field	Ultra (Fitbit)	5 over left pants pocket, 5 over right pants pocket	Steps/min, steps/hour, steps/day	10 devices collected movement (yes vs no) across minutes (98 %); two-way median ICC of absolute agreement 0.90 (steps/min), 1.00 (steps/hour), 1.00 (steps/day); concordance CC 0.90 (steps/min), 1.00 (steps/hour), 0.99 (steps/day); from Bland-Altman plots 95 % of the measures were within the boundaries of 28 steps above and below the mean difference; maximum difference for all devices was 3.3 %
Mammen (2012) [36]	1	0	6 trials were performed while the researcher wore the devices and walked 20 steps	Lab	Ultra (Fitbit)	3 trials on right hip, 3 trials on left hip	Steps/trial	All trackers were within +/−5 % of each other
Meltzer (2015) [37]	9	Not reported	1 night's sleep	Lab	Ultra (Fitbit)	2 on nondominant wrist	TST, sleep efficiency	Among n = 7: no differences between trackers for TST (468.7 vs. 471.1 min normal mode; 300.4 vs. 289.9 min sensitive mode) or sleep efficiency (92.9 % vs. 93.3 % normal mode; 59.4 % vs. 57.4 % sensitive mode)
Montgomery- Downs (2012) [38]	3	Not reported	1 night's sleep	Lab	Classic (Fitbit)	2 on nondominant wrist	Sleep vs. wake	3 participant's recorded 96.5 %, 99.1 %, and 97.6 % agreement at 1-minute epochs
Takacs (2014) [43]	30	50	5 min each of treadmill walking (0.90, 1.12, 1.33, 1.54, 1.78 meters/second)	Lab	One (Fitbit)	1 on the waist at each hip, 1 in front pocket of the dominant leg	Steps/trial, distance/trial	Across 5 treadmill speeds ICC: range 0.95-1.00 (steps), range 0.90-0.99 (distance)

Abbreviations: *CC* correlation coefficient, *EE* energy expenditure, *ICC* intraclass correlation coefficient, *min* minute, *mph* miles per hour, *TST* total sleep time

### Reliability for steps

Comparing two different hip-worn trackers for 16 to 23 participants during treadmill walking and running, the intraclass CC was substantial to almost perfect for steps taken for the Classic (range 0.86–0.91) and the Ultra (range 0.76–0.99) [29]. In another study, during six treadmill walking trials of 20 steps by one researcher, three hip-worn Ultras were compared and all trackers read within 5 % of each other [36]. In a field-based study of 10 hip-worn Ultras all worn by the same person at the same time for 8 days, the median intraclass CC was 0.90 for steps/minute, 1.00 for steps/hour, and 1.00 for steps/day, and comparing across trackers, the maximum difference was only 3.3 % [32].

Comparing three hip-worn Ones worn by 23 participants during treadmill walking and running, the Pearson CC between the left and right hip, as well as both right hips, was almost perfect for steps (0.99 and 0.99, respectively) [15]. In another study, 30 participants wore three Ones on their hips and front pants pocket while walking or running at five different speeds on the treadmill and correlation for steps was almost perfect when comparing across trackers (intraclass CC 0.95–1.00) [43]. Lastly, comparing two wrist-worn Flex trackers worn by 23 participants during treadmill walking and running, the Pearson CC between the left and right wrist was almost perfect for steps (0.90) [15].

### Reliability for distance

In the only study of reliability assessment of distance, 30 participants wore three Ones on their hips and front pants pocket while walking or running at five different speeds on the treadmill and the correlation was almost perfect for distance measurements across trackers (intraclass CC 0.90–0.99) [43].

### Reliability for energy expenditure

Comparing two different hip-worn trackers for 16–23 participants during treadmill walking and running, the intraclass CC was substantial to almost perfect for kilocalories expended for the Classic (range 0.74–0.92) and the Ultra (range 0.91–0.97) [29]. Comparing three hip-worn Ones worn by 23 participants during treadmill walking and running, the Pearson CC between the left and right hip, as well as both right hips, was almost perfect for kilocalories expended (0.97 and 0.96, respectively) [15]. These same participants wore two Flex trackers on their wrists during treadmill walking and running that had almost perfect correlation for kilocalories expended (0.95) [15].

### Reliability for sleep

Three participants wore two Classics overnight and recorded almost perfect levels of agreement (96.5–99.1 %) to classify whether the minute-level data was a sleep or wake minute [38]. Similarly, nine youth participants wore two Ultras on their wrist overnight, with data available for seven participants (one pair did not record and one pair had significant discrepancies between readings) [37]. They found similar readings for total sleep time and sleep efficiency for either the normal or sensitive mode.

#### Feasibility

Feasibility assessment was abstracted for the 22 studies in this review. In total, seven of 18 studies reported on missing or lost data, with the lab-based studies less likely to report it than the field-based studies. For the lab measurements, Case et al. [30] indicated 1.4 % of data were missing from all tested trackers due to not properly setting them to record steps, Dannecker et al. [31] indicated incomplete data on two of 19 participants, and Gusmer et al. [34] excluded six of 32 participants because ActiGraph step counts were about half of the Ultra step counts (they note this is most likely an ActiGraph failure). For one night of recording in the sleep laboratory, Meltzer et al. [37] reported missing data for 14 of 63 participants to assess validity, due to data not recording for the Ultra (*n* = 12) and corrupted PSG files (*n* = 2).

For a field-based study of 21 participants during 2 days of wear some data were lost: moderate-to-vigorous physical activity (*n* = 7 due to data extraction of the One and the Zip (i.e., certain data were only available for a limited amount of time), *n* = 1 Zip malfunction), steps (*n* = 1 Zip malfunction), energy expenditure (*n* = 1 Zip malfunction), and sleep (*n* = 2 participant error for the One) [33]. In a second field-based study enrolling adults > =60 years of age, authors excluded five of 15 participants because they had difficulty with the Classic over the 10-day period (two lost the tracker and three failed to plug it into the wireless base to transmit data) [41]. In a separate field-based study, the Zip was worn over 1 week and five of 47 participants had at least some missing data [44].

## Discussion

This review summarized the evidence for validity and reliability of activity trackers, identifying 22 studies published since 2012. While conducting this review, we learned how the trackers can be set-up to improve upon off-the-shelf accuracy. Those testing and wearing the trackers are encouraged to consider several tips to potentially improve the trackers’ performance (Table 6).

**Table 6:** Strategies to improve the activity tracker accuracy for steps, distance, physical activity, energy expenditure, and sleep

Instruction	Explanation	Web Links: accessed 10/14/2015
Wear the tracker in the same position each day	While wearing the activity tracker in the same position daily may be obvious for the wrist-based trackers, those worn on a pocket, bra, or hip could vary in accuracy depending on location. Trackers are more accurate when worn close to the body^a^. For free-living research studies, the wearing location should be standardized and communicated to participants.	^a^ http://help.fitbit.com/articles/en_US/Help_article/How-do-I-wear-my-Zip/
Enter your details and sync	At initial set-up, users should accurately enter height, weight, gender, and age into the application and sync it to the tracker. For example, these characteristics, as well as heart rate if available, are used by the Fitbit to calculate energy expenditure^b^. Related to this, if body weight meaningfully changes, then updating the tracker with the new weight would be important.	^b^ http://help.fitbit.com/articles/en_US/Help_article/How-does-Fitbit-know-how-many-calories-I-ve-burned
For wrist-worn trackers, indicate if wearing it on the dominant or non-dominant side	In the software set-up, indicate if possible whether the wrist-worn tracker is being worn on the dominant or non-dominant hand. For Jawbone, trackers worn on the non-dominant wrist may be more accurate^c^, probably because the non-dominant hand is less active than the dominant hand, so it provides a better representation of overall body movement. Fitbit indicates that using the non-dominant hand setting increases sensitivity of step counting and can be used if the tracker is under counting steps^d^.	^c^ https://jawbone.com/up/faq
		^d^ http://help.fitbit.com/articles/en_US/Help_article/How-accurate-is-my-Surge
Calibrate stride length	Calibrating stride length may improve distance measures. In our review, only one study indicated that this was performed [34]. Fitbit indicates a default stride length is used otherwise, based on height and gender^e^. Jawbone also provides information for calibration^f^.	^d^ http://help.fitbit.com/articles/en_US/Help_article/How-do-I-measure-and-adjust-my-stride-length
		^e^ https://help.jawbone.com/articles/en_US/PKB_Article/424
Use add-on features and obtain updates	Using add-on features and obtaining updates might become more important since future iterations of algorithms to calculate physical activity or energy expenditure may use new features, such as heart rate and respiration. For example, Fitbit indicates that trackers with heart rate better recognize “active minutes” for physical activities that do not incorporate stepping, such as weight lifting or rowing^e^.	^f^ https://help.fitbit.com/articles/en_US/Help_article/What-are-very-active-minutes/
Add more information via the diary or journal function	Providing information to the tracker on the specific physical activity being performed can help the tracker learn what activities look like for the individual. This is particularly important if the algorithms used by the activity tracker rely on machine learning techniques.	
Interact with the sleep mode settings	Interacting with the sleep mode settings may help the tracker learn if the user is sleeping, napping, or awake. Fitbit indicates that the normal mode counts significant movements as being awake and is appropriate for most users, while the sensitive setting will record nearly all movements as time awake^f^.	^g^ http://help.fitbit.com/articles/en_US/Help_article/Sleep-tracking-FAQs#Whatisthedifference

These options may not be available for all trackers that were reviewed

### Validity and reliability

From this review, we found the validity (Fitbit and Jawbone) and interdevice reliability (Fitbit) of steps counts was generally high, particularly during laboratory-based treadmill tests. When errors were higher, the direction tended to be an under-estimation of steps by the tracker compared to the criterion. This may be particularly problematic at slow walking speeds, similar to findings when testing pedometers [51]. Specifically for steps, if the option is available to set stride length, this should improve accuracy (Table 6). Hip-worn trackers generally performed better at counting steps than trackers worn elsewhere on the body, although Mammen et al. [36] suggests moving the placement from the hip if being worn by an older adult with slower gait speed. Only one study assessed the validity and reliability of distance walked, finding that while reliability was high, distance was over-estimated at slower speeds and under-estimated at faster speeds [43].

Compared to other accelerometers, one study indicated that the trackers generally over-counted moderate-to-vigorous physical activity, with some large differences found (mean 0.3, 1.0, and 1.5 h/day for the UP, One, and Zip, respectively) [33]. However, another study indicated higher agreement [44]. It may be that the cutpoints [49] used to define moderate-to-vigorous physical activity in both studies were set too high, particularly for older or inactive adults. The reliability of physical activity measurement has not been tested in any study.

From 10 adult studies, we found that although interdevice reliability of energy expenditure was high, the validity of the tracker was lower. When reported, the CC generally ranged from moderate to substantial agreement. Across trackers, many studies indicated that the bias in mis-reporting was often an under-estimation of energy expended.

For sleep among youth and adults, despite high reliability, the trackers evaluated generally over-estimated total sleep time [33, 37, 38, 47, 48], and when tested against PSG the trackers over-estimated sleep efficiency and under-estimated wake after sleep onset [37, 38, 47, 48]. These findings are similar to other studies of accelerometry, in which the devices are highly sensitive but do not accurately detect periods of wake before and during sleep [52]. However, for one tracker the sensitive mode setting was tested, which under-estimated total sleep time and sleep efficiency and over-estimated wake after sleep onset [37]. Work is needed to improve the validity of sleep measurement with these trackers, particularly when using them for only one or two nights of testing [38]. It may be that newer trackers will perform better if they “learn” when the person is asleep, awake, or napping (Table 6).

### Feasibility

Seven of 22 studies reported on missing or lost data, ranging from approximately 1.4 to 22.2 % for laboratory-based studies and 10.6 to 33.3 % for field-based studies. Some of the lost data was attributable to the validation criterion measure and not the trackers, and other lost data were attributable to researcher error and not participant error. Even so, researchers should anticipate data loss based on these findings. Future studies should report missing data and the reason for the loss. One study in this review [44] and others not included [4, 8, 19, 53] report relatively high acceptability in wearing the trackers. This type of information may help with understanding reasons for missing data in field-based studies, particularly if they occur over long time periods.

### For the companies

Through this review, we identified three recommendations manufacturers can contribute to enhance the use of the trackers for research. First, the trackers contain firmware, defined as an electronic component with embedded software to control the tracker. Firmware can be updated by the company at any time; when the tracker is synched, the new software is updated. These software changes can influence the measurement properties in either positive or negative ways, and can change what might have been previously confirmed or published. Firmware may fix bugs or add features to the tracker, or it may change how variables are calculated. However, many other changes take place, which the consumer cannot detect [54]. As an alternative, the company supporting ActiGraph accelerometers currently makes firmware updates available to the public via their website, allowing researchers to assess those changes for impact on the measurement properties of the accelerometer [55, 56]. A similar standard operating procedure would be a beneficial approach for researchers using these trackers.

Second, Jawbone UP3 and UP4 trackers include bioelectric impedance, with corresponding measures of heart rate and respiration, and both skin and ambient temperatures. Additionally, some of the newer Fitbit trackers include GPS (Surge) and optical heart rate sensors (Surge and Charge HR). With these enhancements, the companies seemingly have the tools to determine whether the tracker is being worn (e.g., adherence) and whether it is being worn by the same individual (e.g., one body authentication) [8]. It would be beneficial if the companies derived an indicator of wear and made this available on a minute-by-minute level, corresponding to other available data. Currently, neither the Jawbone nor Fitbit indicate the time worn, which could impact all metrics studied in this review.

Third, the companies could allow access to more data that are collected. At present, the trackers provide users with only a subset of data that is actually collected. The companies control the output available, making the day-level summary variables the easiest to obtain. For example, despite capturing GPS and heart rate on two trackers, Fitbit currently limits the export of these full datasets. Furthermore, the resulting output is derived through proprietary algorithms that may change over time and with new features. In all likelihood, based on the performance of the trackers found in this review, these algorithms are supported through machine learning techniques. At a minimum, it would be helpful for companies to reveal what pieces of data are being used by the trackers to calculate each output measure. For example, Jawbone indicates that height, weight, gender, age, and heart rate, if available, are used to calculate physical activity [14].

### Future research

In total, Fitbit offered at least 9 trackers since 2008 and Jawbone offered at least 6 trackers since 2011. Until we understand if the specifications within a company’s family of trackers are similar, researchers should confirm the validity and reliability of new trackers. Moreover, an argument could be made to test any new tracker, even if the company confirms similar hardware and software processes. With time, the trackers offer more features through enhancements made to the trackers (Table 1). Each new tracker feature needs testing for reliability, validity, and usability. Specific types of activities should also be tested, similar to the study by Sasaki et al. [39]. While this review focused on steps, distance, physical activity, energy expenditure, and sleep, other features to test include number of stair flights taken, heart rate, respiration, location via GPS technology, skin temperature, and ambient temperature.

Exploring the measurement properties of the trackers in a wide variety of populations would also be important in both laboratory and field settings. Free-living activities may better reflect the true accuracy of the tracker, because daily activities include a considerable amount of upper body movement that may or may not be accurately captured by the trackers [35]. Currently, the review only identified two studies that included children [37, 48]. Researchers mostly tested the trackers in middle-aged adult populations with normal BMI. Since studies of pedometers indicate lower accuracy among participants with higher BMI [57], it would be prudent to test various trackers types and locations among participants with higher BMI [43].

Moreover, with the proliferation of trackers, researchers would benefit from an evidence-based position statement on the properties necessary to consider a tracker valid and reliable [38]. Guidance on equivalency of accelerometers exists [58], but this review found a variety of statistical methods applied to the data and interpreted slightly differently across studies. Those who conduct future studies on the measurement properties of the trackers should be sure to initialize the tracker properly and indicate in the publication how this was done so others can replicate the process. Providing the specific tracker type, date purchased, and date tested would also be important.

Notably there were no reliability studies of any Jawbone tracker or the Fitbit Zip, and no intradevice reliability studies of any trackers. While more field-based studies are needed, the laboratory studies indicated high interdevice reliability for measuring steps, energy expenditure, and sleep. Only one study assessed distance, also finding high interdevice reliability during treadmill walking and running [43]. It would be ideal practice for all studies or programs to test the trackers for reliability before deploying them for either measurement or intervention.

While not reviewed here, researchers should also consider issues related to privacy and informed consent with activity trackers and smart phone applications [59, 60]. Since the trackers can measure and store data for long periods of time passively, providing informed consent takes on new meaning with the extended time period, locational information, and re-use of data in successive analyses. Users should also be aware that the companies access and use the data that are entered and collected [61]. Recent examples include an indication of the states with the most steps by Fitbit users [62] and the impact of the prior day’s sleep and steps taken on self-reported mood by Jawbone users [63].

### Limitations

Our review has several limitations. The literature on activity trackers is rapidly building and it is possible that studies were missed despite our best efforts. We encountered some challenges with comparing across studies, due to varying methods and reported results. The findings should be viewed in light of the variety of study protocols and methodology.

When we began the systematic review in fall 2014, we were guided by the most recent market data available at that time, indicating that Fitbit and Jawbone represented the majority of the consumer market [2]. In June 2015, market share from the first quarter sales in 2015 indicated the top five vendors were Fitbit (34 %), Xiaomi (25 %), Garmin (6 %), Samsung (5 %), and Jawbone (4 %) [64]. There is a built-in time lag between manufacturing and sale of activity trackers to use in the research laboratory and field. Thus, some activity trackers that are currently available to consumers were not represented in this review, but should be considered as future studies accumulate on new devices and brands.

## Conclusions

This systematic review of 22 studies included assessments of five Fitbit and two Jawbone trackers, focusing on validity and reliability of steps, distance, physical activity, energy expenditure, and sleep. No single specific tracker had a complete assessment across the five measures. This review also described several ways to improve the trackers’ accuracy, offered recommendations to companies selling the trackers, and identified future areas of research. Generally, the review indicated higher validity of steps, fewer studies on distance and physical activity, and lower validity for energy expenditure and sleep. These studies also indicated high interdevice reliability for steps, energy expenditure, and sleep for certain Fitbit models, but with no studies on the Jawbone. As new activity trackers and features are introduced to the market, documentation of the measurement properties can guide their use in research settings.

## Additional file

Additional file 1:
**Flow of article selection using the PRISMA schematic (Liberati et al., 2009 [** 27 **]; Moher et al., 2009 [** 28 **]).** (PDF 62 kb)

## Abbreviations

BMI: Body mass index
CC: Correlation coefficient
GPS: Global positioning system
PRISMA: Preferred Reporting Items for Systematic Reviews and Meta-Analyses
PSG: Polysomnography
SD: Standard deviation
US: United States
