# Supplementary material for: Systematic review of the validity and reliability of consumer-wearable activity trackers
Source: Int J Behav Nutr Phys Act. 2015 Dec 18;12:159. doi: 10.1186/s12966-015-0314-1 (PMC4683756; doi:10.1186/s12966-015-0314-1)

Additional File 1: Flow of article selection using the PRISMA schematic (Liberati et al., 2009; Moher et al., 2009)

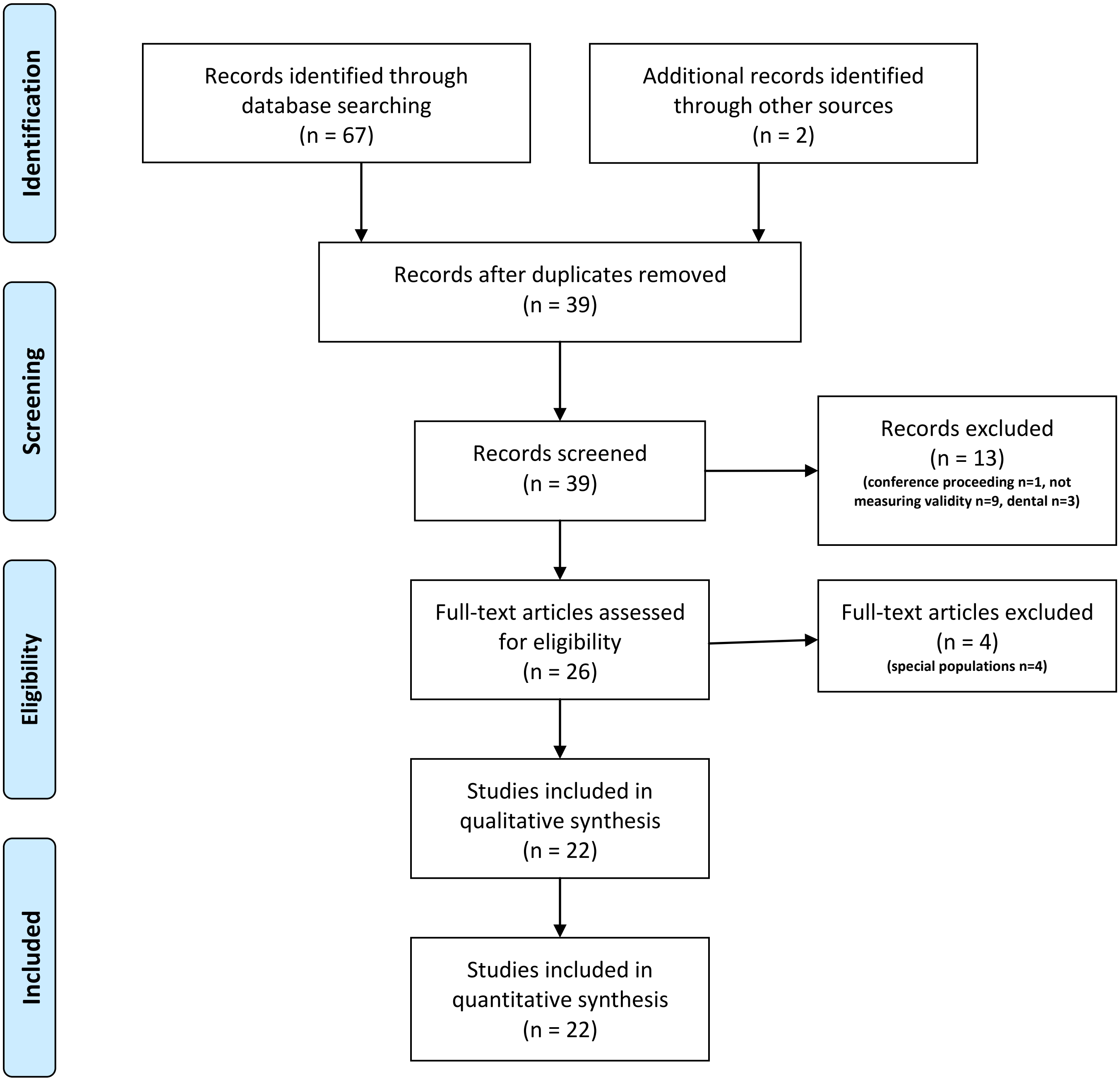

Supplement: Additional file 1: — Flow of article selection using the PRISMA schematic (Liberati et al., 2009 [ 27 ]; Moher et al., 2009 [ 28 ]). (PDF 62 kb) [file 12966_2015_314_MOESM1_ESM.pdf]
